# Integrative identification of immune-related key genes in atrial fibrillation using weighted gene coexpression network analysis and machine learning

**DOI:** 10.3389/fcvm.2022.922523

**Published:** 2022-07-27

**Authors:** Peng-Fei Zheng, Lu-Zhu Chen, Peng Liu, Zheng-Yu Liu, Hong Wei Pan

**Affiliations:** ^1^Department of Cardiology, Hunan Provincial People's Hospital, Changsha, China; ^2^Clinical Research Center for Heart Failure in Hunan Province, Changsha, China; ^3^Hunan Provincial People's Hospital, Institute of Cardiovascular Epidemiology, Changsha, China; ^4^Department of Cardiology, The Central Hospital of ShaoYang, Shaoyang, China

**Keywords:** weighted gene coexpression network analysis, atrial fibrillation, immune cell subtype distribution pattern, machine learning, hub genes

## Abstract

**Background:**

The immune system significantly participates in the pathologic process of atrial fibrillation (AF). However, the molecular mechanisms underlying this participation are not completely explained. The current research aimed to identify critical genes and immune cells that participate in the pathologic process of AF.

**Methods:**

CIBERSORT was utilized to reveal the immune cell infiltration pattern in AF patients. Meanwhile, weighted gene coexpression network analysis (WGCNA) was utilized to identify meaningful modules that were significantly correlated with AF. The characteristic genes correlated with AF were identified by the least absolute shrinkage and selection operator (LASSO) logistic regression and support vector machine recursive feature elimination (SVM-RFE) algorithm.

**Results:**

In comparison to sinus rhythm (SR) individuals, we observed that fewer activated mast cells and regulatory T cells (Tregs), as well as more gamma delta T cells, resting mast cells, and M2 macrophages, were infiltrated in AF patients. Three significant modules (pink, red, and magenta) were identified to be significantly associated with AF. Gene enrichment analysis showed that all 717 genes were associated with immunity- or inflammation-related pathways and biological processes. Four hub genes (*GALNT16, HTR2B, BEX2*, and *RAB8A*) were revealed to be significantly correlated with AF by the SVM-RFE algorithm and LASSO logistic regression. qRT–PCR results suggested that compared to the SR subjects, AF patients exhibited significantly reduced *BEX2* and *GALNT16* expression, as well as dramatically elevated *HTR2B* expression. The AUC measurement showed that the diagnostic efficiency of *BEX2, HTR2B*, and *GALNT16* in the training set was 0.836, 0.883, and 0.893, respectively, and 0.858, 0.861, and 0.915, respectively, in the validation set.

**Conclusions:**

Three novel genes, *BEX2, HTR2B*, and *GALNT16*, were identified by WGCNA combined with machine learning, which provides potential new therapeutic targets for the early diagnosis and prevention of AF.

## Introduction

Characterized as rapid disordered atrial electrical activity, atrial fibrillation (AF) was defined as a common persistent arrhythmia in the clinic (1). Based on an existing report, almost 1% of the population is affected by AF worldwide, and its prevalence is positively correlated with age. The incidence of AF in the population older than 80 years reaches 8% (2). AF is significantly correlated with the occurrence of myocardial infarction, stroke, and heart failure, which increases the economic burden not only on the patients' families but also on society (3). Thus, clarifying the pathogenesis and discovering effective therapeutic methods are urgent endeavors. AF is a multifactorial complex disease and is commonly associated with many factors, such as sex, smoking, age, hypertension, diabetes, obesity, valvular heart disease, and ischaemic heart disease (4). However, the exact changes in etiology and pathology in AF are totally unknown. Increasing evidence suggests that immune cells significantly participate in the processes of AF pathogenesis (5). Additionally, a relatively high production of inflammatory markers in the serum of AF patients was observed, such as interleukin 6 (IL-6) and C-reactive protein (CRP) (6, 7). However, to identify potential targets for AF treatment, the connections between immune cells and the molecular pathogenesis mechanisms of AF need to be further explored.

In recent years, the use of CIBERSORT, as a widely used analysis tool, has been applied to RNA-seq data or microarray data to investigate the immune cell infiltration patterns and evaluate the infiltrated immune cell proportions in samples (8). With the continuous promotion of gene chip technology, weighted gene coexpression network analysis (WGCNA), as a powerful biological tool to analyze network relationships and molecular mechanisms, is widely used for massive gene profile analysis (9). WGCNA is often employed to identify coexpressed gene modules and further explore the connections of these identified gene modules with the features of the sample (10). Recently, an increasing number of researchers have applied machine learning to improve the prediction and accuracy of these genes, which were identified through traditional microarrays or next-generation sequencing data (11). The SVM-RFE algorithm and LASSO regression are the most widely used machine learning methods to identify key genes (12). However, the combined application of WGCNA and machine learning in the identification of AF-associated genes has not been conducted.

In the current research, comprehensive bioinformatics analysis was conducted to investigate the association of immune-associated genes and cells with AF. The composition of the immune cells infiltrated in the tissues was assessed using CIBERSORT, the key gene modules were identified by WGCNA, and the key genes correlated with AF were identified *via* the SVM-RFE algorithm and LASSO regression. Furthermore, the expression of key genes and their diagnostic efficiency were further validated in the training set and validation samples.

## Materials and methods

### Collection of datasets

Gene expression profiles of left atrial samples of GSE79768 (AF patients = 7, SR individuals = 6) and GSE115574 (AF patients = 14, SR individuals = 15) were extracted from the public Gene Expression Omnibus (GEO, http://www.ncbi.nlm.nih.gov/geo) database. The integrated expression profiles of GSE79768 and GSE115574 were used as training sets. Gene expression profiles were normalized using the *normalize Between Arrays* function in the *limma* package (13). Probes that detected more than one gene were excluded from this study. The expression of genes detected by multiple probes was determined as the average gene expression detected for all probes. Interbatch differences between the GSE79768 and GSE115574 datasets were eliminated using the “sva” package.

### Construction of the WGCNA and identification of modules significantly associated with AF

A critical tool in the study of systems biology is WGCNA, which can construct a gene expression data profile-based scale-free network (14). The WGCNA method was used to analyze the top 25% of genes with high expression variances. The reliability of the constructed scale-free network is ensured by removing outlier samples. First, before the power function was applied, a standard-scale free network was used to approximate the appropriate soft threshold power (soft power = 10) to obtain adjacency values among genes with a variance more significant than all the variance quartiles. Next, we transformed the adjacency values into a topological overlap matrix (TOM) and derived the dissimilarity (1-TOM) values. Finally, the dynamic tree cut method was used to identify modules by hierarchically clustering genes with the 1-TOM as the distance measure with a deep split value of 2 and a minimum size cut-off of 100 for the resulting dendrogram. To evaluate the relationships between clinical shapes and modules for discerning modules of biological significance, we conducted Pearson correlation analysis.

### Enrichment analysis of interesting modules

KEGG and GO enrichment analyses of genes in biologically significant modules were carried out by clusterProfiler and the DOSE package in R (15). The threshold was determined to be FDR < 0.05.

### Identification of key genes by LASSO regression and SVM-RFE algorithm

The LASSO regression and SVM-RFE algorithm were used to identify the key genes with the best prognostic value for AF. LASSO regression (16) was carried out with the package “glmnet”. As a technique for effective feature selection, SVM-RFE can select the best variables by excluding the SVM-generated feature vector (17). Based on the SVM function in the e1071 package of R, the selected biomarkers in the diagnosis of AF were classified and analyzed by the SVM classifier. The common genes identified by these two machine learning methods were defined as key genes for subsequent research.

### Evaluation of immune cell subtype distribution

The immune infiltration pattern in AF was explored using a CIBERSORT R script (8). After downloading the immune cell expression matrix, boxplot diagrams, heatmaps, and histograms were generated using the package “ggplot2”. The histogram shows the immune cell proportion infiltrated in AF patients, and the heatmap and boxplot diagrams show the difference in immune cell infiltration in control and AF subjects. The Pearson correlation coefficient between each immune cell was calculated using the package “corrplot”, and the results are shown in the relevant heatmap.

### Correlation between key genes and immune cells and roc curve analysis in training set

The “corrplot” software package was employed to generate the Pearson correlation coefficient between each immune cell and hub gene, and the results are shown in the relevant bar graph. The diagnostic accuracy of the key genes was also tested in the training set.

### Study population

A total of 158 participants, including 82 persistent AF patients and 76 SR subjects, were recruited from Hunan Provincial People's Hospital from June to December 2021. The disease was continuously sustained for more than 7 days or more than 7 days after cardioversion (automatic, electrical, or drug cardioversion) and was defined as persistent AF (18). Patients with a history of haematologic disease, type 1 diabetes, coronary artery disease, hypertension, autoimmune disease, neoplasia, and renal or liver diseases were excluded. Peripheral blood samples were collected and placed at −80°C for subsequent study. Study protocols were developed based on the Ethics Committee of Hunan Provincial People's Hospital (No: LL-20210615-144) and the 2008 revision of the Declaration of Helsinki of 1975 (http://www.wma.net/en/30publications/10policies/b3/). All subjects provided written and informed consent.

### qRT-PCR

Total RNA was extracted from peripheral blood with an RNeasy™ Mini Kit (QIAGEN, Frankfurt, Germany). cDNA was then reverse-transcribed with the PrimeScript™ RT reagent Kit (Takara, Otsu, Japan). A Taq PCR Master Mix Kit (Takara, Otsu, Japan) was used to perform qRT–PCR using an ABI 7500 instrument (Applied Biosystems, USA). The proprietary qPCR primers used in the experiment were designed and validated by Songon Biotech (Songon Biotech, Shanghai, China).

### Statistical analyses

All the data displayed in this study were processed and analyzed with SPSS (Version 22.0). *GALNT16, HTR2B, RAB8A*, and *BEX2* expression were assessed using independent sample t tests. According to the concentrations of *GALNT16, HTR2B, RAB8A*, and *BEX2* in serum, we constructed receiver operating characteristic (ROC) curves. A nonparametric method was employed to evaluate the respective areas under the curves (AUCs) with 95% CI by MedCalc (MedCalc Software, Mariakerke, Belgium, version 19.7.4) software. R (version 4.1.0) was used to perform the bioinformatics analysis. *P* < 0.05 was considered statistically significant.

## Results

### Data preprocessing

First, the normalized gene expression profiles of the GSE79768 and GSE115574 datasets were obtained after standardizing the data format, adding missing values, and removing outliers. Then, after data merging and eliminating the interbatch differences between the GSE79768 and GSE115574 datasets, the combined expression matrix including 21,629 gene symbols was obtained from the 42 left atrial samples in the training set. After removing 1 outlier sample (Supplementary Figure S1), the top 25% of genes with high expression variance in the remaining 41 left atrial samples were selected for subsequent WGCNA and are presented in Supplementary Table S1. In addition, disease grouping information for 41 samples is also presented in Supplementary Table S2.

### Weighted gene co-expression networks

**Figure 1:**
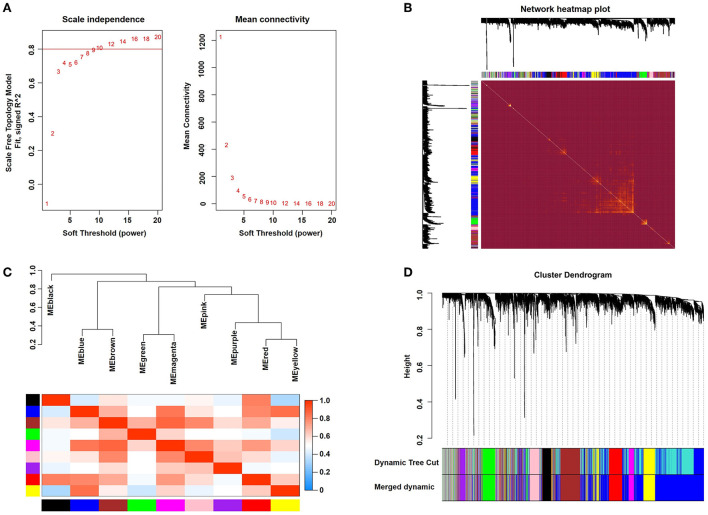
Weighted gene coexpression network analysis. **(A)** Analysis of network topology for various soft-thresholding powers. **(B)** Representative heatmap representing the topological overlap of the gene network. **(C)** Correlations among the indicated modules are shown. **(D)** The gene clustering dendrograms are shown.

After calculation, we revealed that a correlation coefficient of more than 0.8 (the soft threshold β is 10) was highly correlated and suitable for constructing several gene modules (Figure 1A). A TOM was constructed by calculating the correlation and adjacency matrices of the gene expression profiles. The gene cluster tree is depicted in Figure 1B. Next, to select each gene network's gene modules, both TOM and hierarchical average linkage clustering were employed. Figure 1C depicts the heatmap. The dynamic tree cut algorithm depicted twelve gene modules (Figure 1D).

### Identification of modules of interest

**Figure 2:**
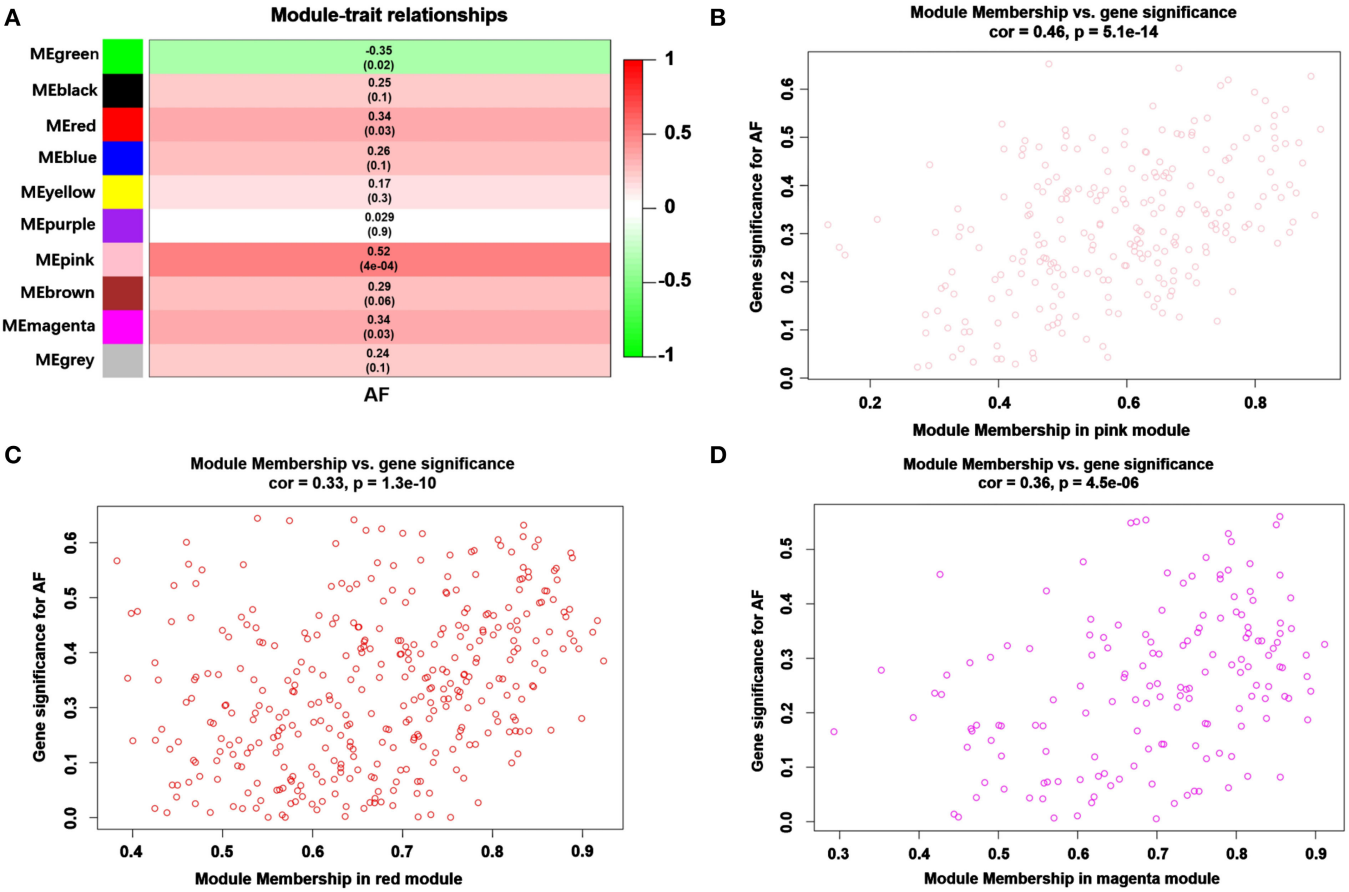
Module-feature associations and associations between gene significance and module membership (MM). **(A)** The module genes are listed in each row, and the clinical phenotype is listed in each column. The correlations and the *p values* for listed genes and clinical phenotypes are shown in each cell. The different correlations are represented as different colors. A representative scatter plot displays the correlations between MM and GS vs. module membership (MM) of AF in the pink **(B)**, red **(C)** and magenta **(D)** modules.

Modules closely related to clinical features are often found to carry important and specific biological significance. As shown in Figure 2A, the pink (*r*^2^ = 0.52, *P* = 4E-04), red (*r*^2^ = 0.34, *P* = 0.03) and magenta (*r*^2^ = 0.34, *P* = 0.03) modules appeared to be positively correlated with AF. An in-depth calculation was performed to discern the association between the color module and gene significance (GS). The association between the pink module and gene significance was 0.46 (*P* = 5.1E-014) (Figure 2B), the association between the red module and gene significance was 0.33 (*P* = 1.3E-10) (Figure 2C), and the association between the magenta module and gene significance was 0.36 (*P* = 4.5E-06) (Figure 2D). All gene symbols in the pink, red, and magenta modules and their GS values and corresponding *P* values are described in detail in Supplementary Table S3.

### Enrichment analysis of genes in pink, brown, and cyan modules

**Figure 3:**
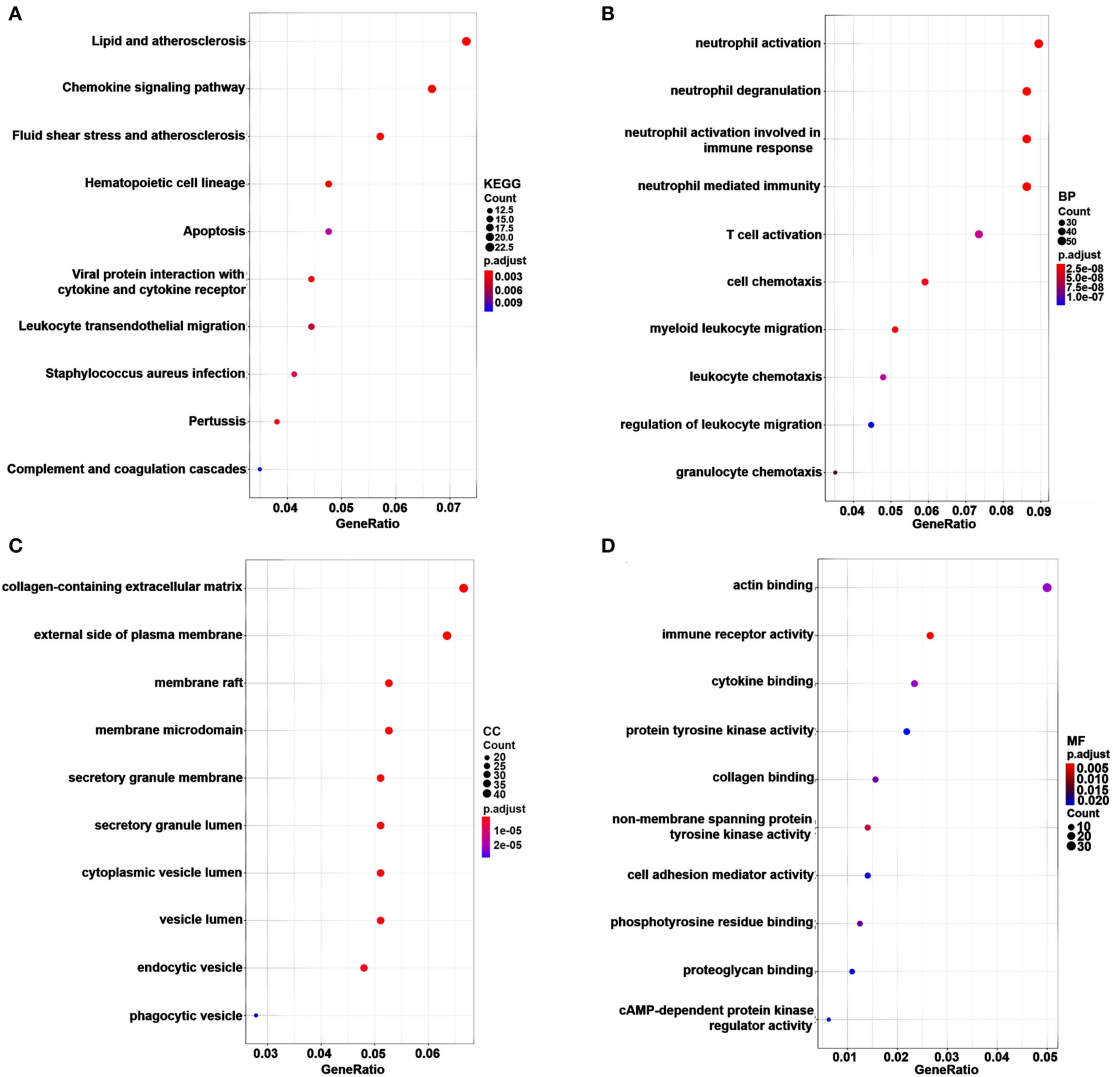
KEGG pathway and GO functional enrichment analyses for genes in the pink, brown and cyan modules. The gene number is represented on the x-axis, and the KEGG pathway and GO terms are presented on the y-axis. **(A)** KEGG pathway. **(B)** Biological process. **(C)** Cytological components. **(D)** Molecular function.

KEGG pathway and GO enrichment analyses of 717 genes in the pink, red, and magenta modules were carried out to dissect their physiological purposes. Figure 3A shows the top 10 KEGG signaling pathways as follows: hsa04640: haematopoietic cell lineage; hsa05418: fluid shear stress and atherosclerosis; hsa05417: lipid and atherosclerosis; hsa04062: chemokine signaling pathway; hsa04061: viral protein interaction with cytokine and cytokine receptor; hsa05133: pertussis; hsa05150: Staphylococcus aureus infection; hsa04670: leukocyte transendothelial migration; hsa04210: apoptosis; hsa04610: complement and coagulation cascades. Figure 3B shows the top 10 biological processes as follows: GO:0042119: neutrophil activation; GO:0043312: neutrophil degranulation; GO:0002283: neutrophil activation involved in immune response; GO:0002446: neutrophil mediated immunity; GO:0097529: myeloid leukocyte migration; GO:0060326: cell chemotaxis; GO:0042110: T-cell activation; GO:0030595: leukocyte chemotaxis; GO:0071621: granulocyte chemotaxis; GO:0002685: regulation of leukocyte migration. These signaling pathways and biological processes are mainly related to inflammation and the immune response. In addition, cytological components and molecular functions are shown in Figures 3C,D. The details of these analyses can also be found in Supplementary Table S4.

### Identification of hub genes

**Figure 4:**
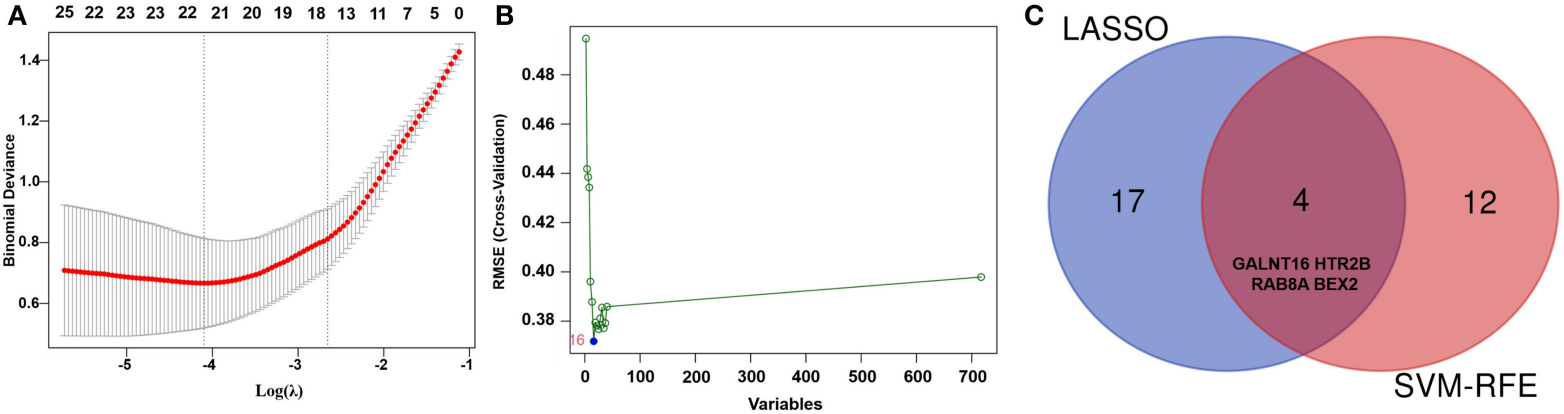
Identification of key genes of AF by machine learning. **(A)** The key genes identified by LASSO regression in AF patients. **(B)** The key genes identified by the SVM-RFE algorithm in AF patients. **(C)** Representative Venn diagram showing the genes extracted using SVM-RFE and LASSO.

To explore reliable serum biomarkers significantly associated with AF, LASSO regression and the SVM-RFE algorithm were implemented to evaluate the characteristic genes in AF according to the gene expression profile in the key modules. The characteristic genes (*n* = 21) were identified by LASSO regression (Figure 4A). Another 16 key genes were identified by the SVM-RFE algorithm (Figure 4B). Then, for subsequent investigations, a total of 4 overlapping genes (*BEX2, GALNT16, RAB8A*, and *HTR2B*) were selected (Figure 4C). In addition, other genes identified with the SVM-RFE algorithm and LASSO regression are listed in Supplementary Table S5.

### Profile of immune cell subtype distribution pattern

**Figure 5:**
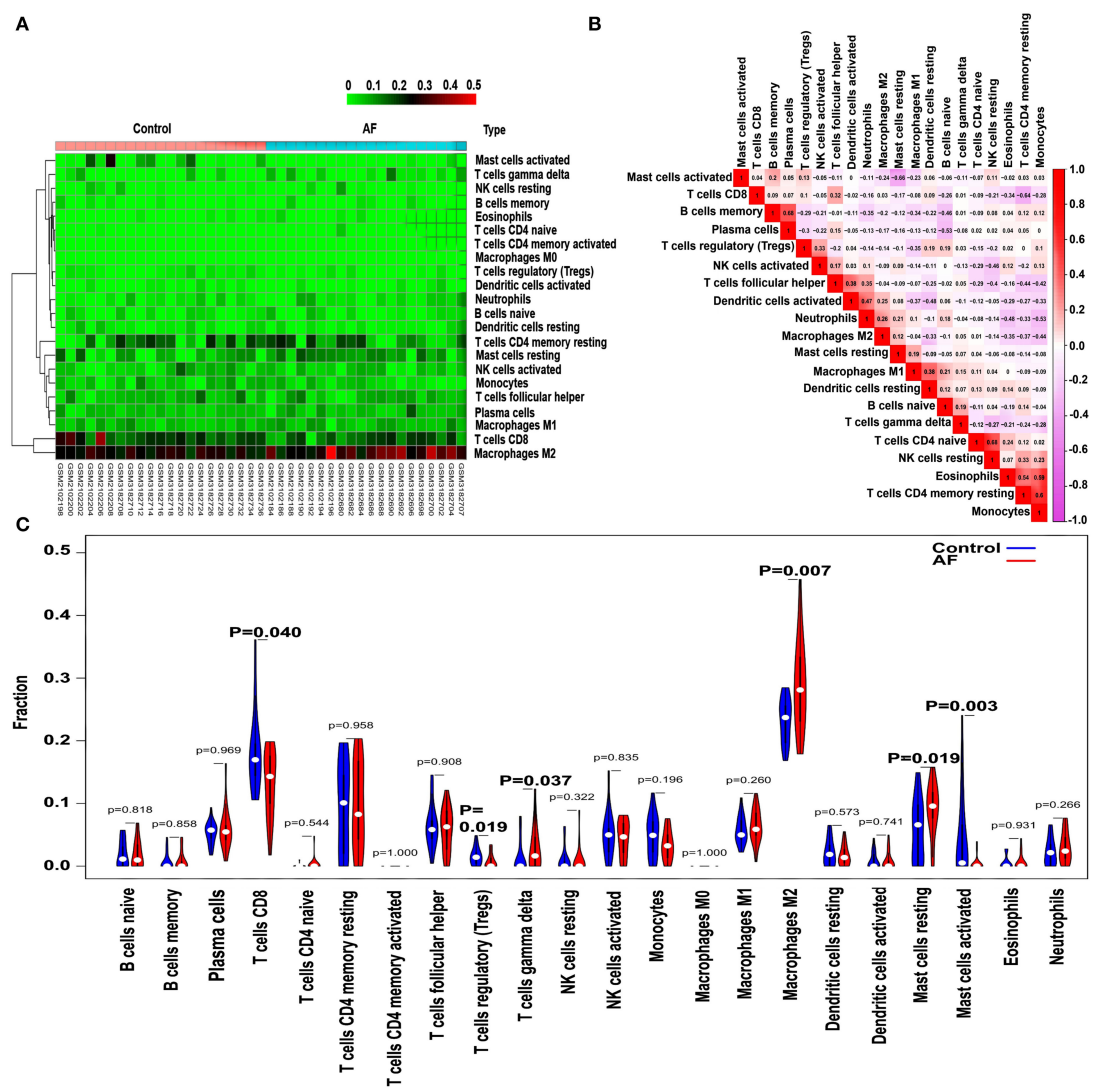
Pattern of immune cell subtype infiltration in the training set. **(A)** Representative heatmap representing the proportions of different immune cells. **(B)** Representative heatmap showing the correlations of the indicated immune cells. **(C)** Representative violin plot showing the different fractions of infiltrated immune cells.

The CIBERSORT algorithm was utilized to evaluate the differential expression of immune fractions between SR and AF samples. The cumulative histogram visually shows the relative proportions of various immune cell subtypes (Supplementary Figure S2). As shown in Figure 5A, significant differences in the immune cell proportions were found between the SR and AF groups of samples. A negative association between activated and resting mast cells was observed using a correlation matrix. Meanwhile, we also observed that Tregs were negatively associated with M1 macrophages and positively associated with activated NK cells; M2 macrophages were negatively associated with resting dendritic cells, eosinophils, monocytes, and resting memory CD4 T cells; CD8 T cells were negatively associated with resting memory CD4 T cells; memory B cells were positively associated with plasma cells (Figure 5B). Compared with SR subjects, AF patients generally exhibited decreased infiltration of activated mast cells and regulatory T cells (Tregs) and increased infiltration of resting mast cells, M2 macrophages, and gamma delta T cells (Figure 5C) (*P* < 0.05-0.01, respectively). In addition, the immune cell infiltration pattern in AF is also shown in Supplementary Table S6.

**Figure 6:**
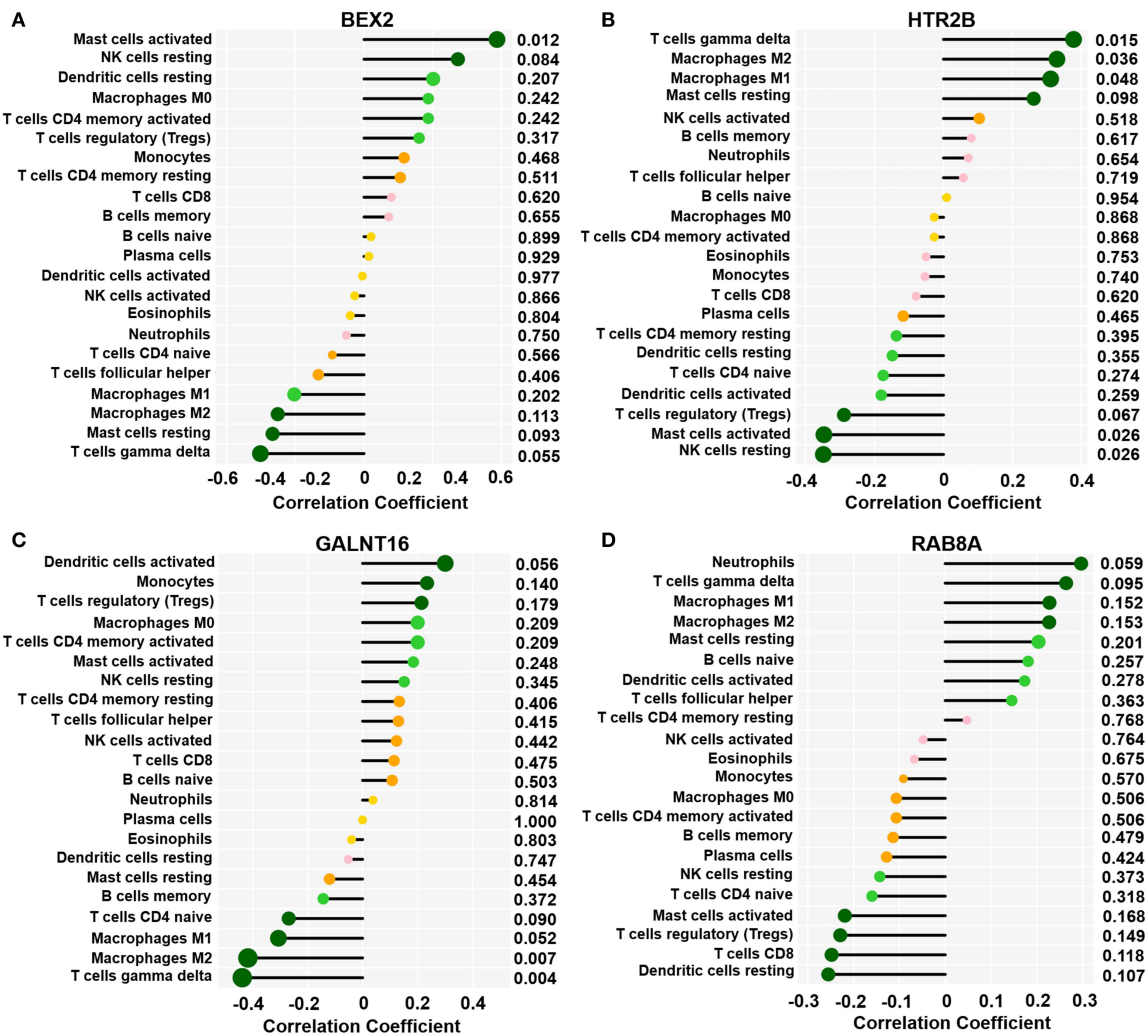
Correlation between key genes and infiltrated immune cells. Representative graphs showing the correlation between the infiltrated immune cells and *BEX2* **(A)**, *HTR2B* **(B)**, *GALNT16* **(C)** and *RAB8A* **(D)**. Correlation strength is shown by the dot size; the p values are expressed as the changes in dot color. *p* < 0.05 represented a significant difference.

As shown in Figure 6, we observed a positive association between the *BEX2* gene and activated mast cells; additionally, the *HTR2B* gene was positively correlated with gamma delta T cells, M2 macrophages, and M1 macrophages and negatively associated with activated mast cells and resting NK cells. In addition, negative correlations between the *GALNT16* gene and M2 macrophages and gamma delta T cells were also observed. However, there was no significant correlation between the *RAB8A* gene and immune cells (*P* < 0.05–0.01).

### Validation of the key genes in AF patients

**Figure 7:**
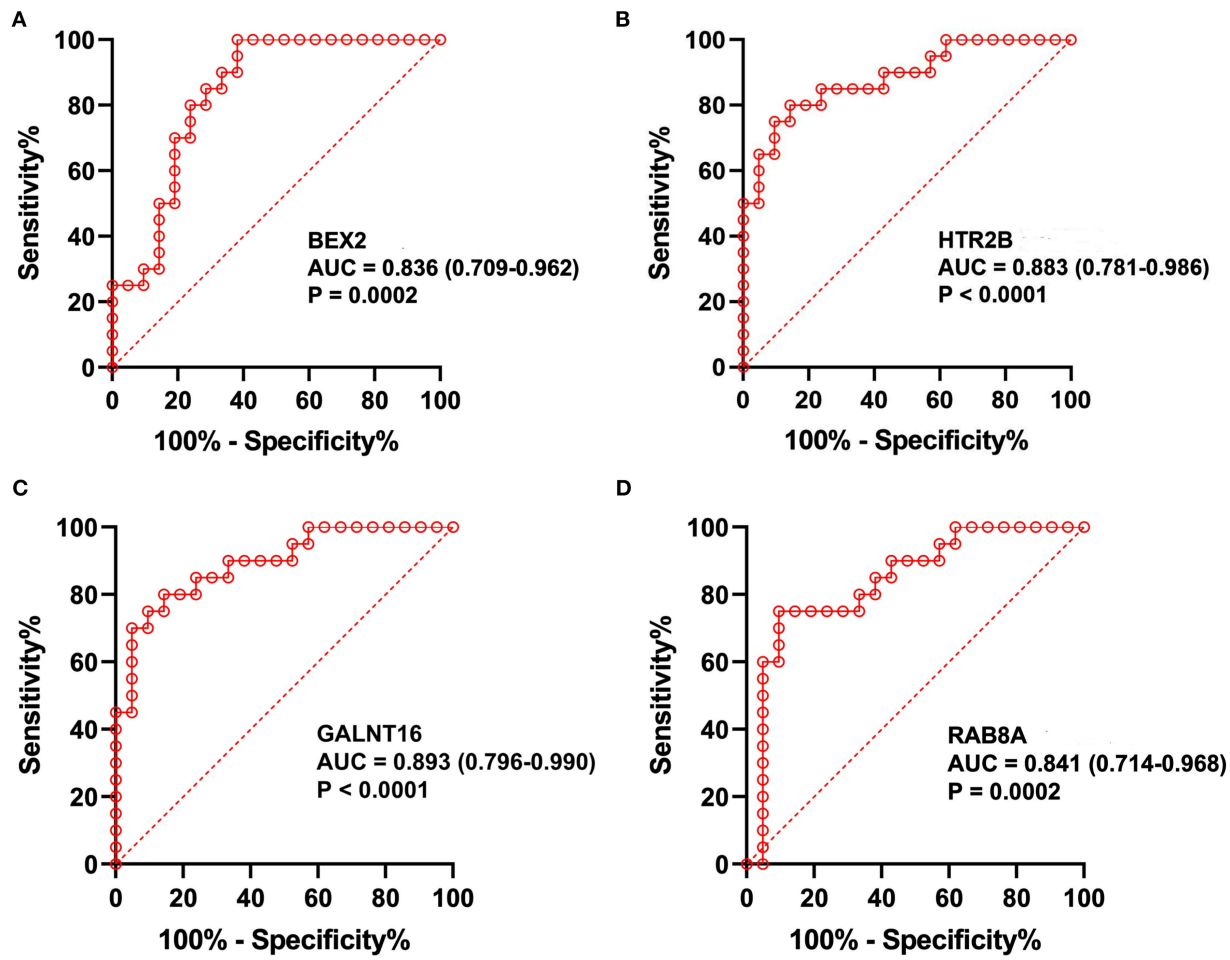
The analysis of ROC curves in the training set. Representative plots showing the results of ROC curve analysis of *BEX2* **(A)**, *HTR2B* **(B)**, *GALNT16* **(C)** and *RAB8A* **(D)** in the training set.

**Figure 8:**
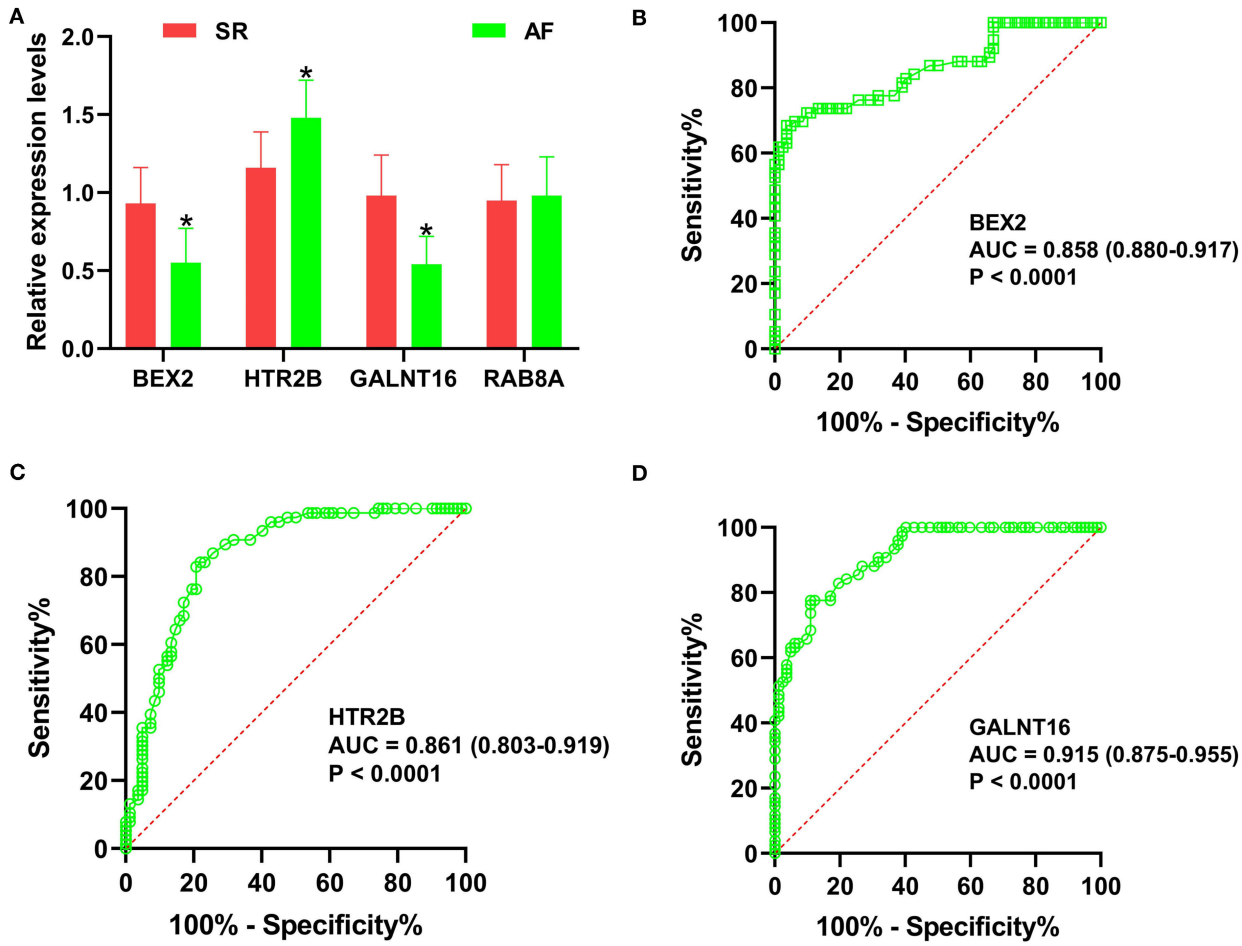
Validation of the key genes in AF samples. **(A)** The relative expression levels of *BEX2, HTR2B, GALNT16* and *RAB8A* in AF patients, *P < 0.01. ROC curve analysis of *BEX2* **(B)**, *HTR2B* **(C)** and *GALNT16* **(D)** in AF patients.

The predictive values of *BEX2, HTR2B, GALNT16* and *RAB8A* for the diagnosis of AF were investigated using ROC curve analysis. The AUC values for *BEX2* (Figure 7A), *HTR2B* (Figure 7B), *GALNT16* (Figure 7C) and *RAB8A* (Figure 7D) were 0.836 (95% CI 0.709–0.962; *P* = 0.0002), 0.883 (95% CI 0.781–0.986; *P* < 0.0001), 0.893 (95% CI 0.796–0.990; *P* < 0.0001) and 0.841 (95% CI 0.714–0.968, *P* < 0.0001) in the training set, respectively. Further verifying the expression of these key genes in AF patients (Figure 8A), we noticed that *BEX2, GALNT16*, and *HTR2B* expression levels were significantly increased in AF patients compared with SR subjects (*P* < 0.01). However, no significant difference in *RAB8A* expression was found between AF patients and SR subjects. Additionally, the AUC values for *BEX2* (Figure 8B), *HTR2B* (Figure 8C), and *GALNT16* (Figure 8D) were 0.858 (95% CI 0.880–0.917; *P* < 0.0001), 0.861 (95% CI 0.803–0.919; *P* < 0.0001) and 0.915 (95% CI 0.875–0.955; *P* < 0.0001), respectively.

## Discussion

In the current research, GSE79768 combined with GSE115574 as training datasets were downloaded from the GEO database and analyzed using WGCNA. Then, three modules (pink, red, and magenta) were identified to be significantly associated with AF. Four hub genes (*GALNT16, HTR2B, RAB8A*, and *BEX2*) were revealed to be significantly correlated with AF by LASSO logistic regression and the SVM-RFE algorithm. The CIBERSORT results suggested decreased infiltration of regulatory T cells (Tregs) and activated mast cells and increased infiltration of resting mast cells, M2 macrophages, and gamma delta T cells in AF patients. qRT–PCR results revealed that *BEX2* and *GALNT16* expression levels were significantly decreased and *HTR2B* expression was significantly increased in AF patients compared with SR subjects. ROC analyses based on the training set and our clinical samples revealed that the *HTR2B, BEX2*, and *GALNT16* genes remained highly effective in distinguishing AF patients from normal SR subjects. Gene enrichment analysis indicated that these key genes were mainly involved in several inflammatory- or immune-related signaling pathways and biological processes.

Through a comprehensive search of the NCBI GENE database, we revealed that brain-expressed X-linked 2 (*BEX2*, also known as BEX1; DJ79P11.1; HGNC: 30933, gene ID: 84707, OMIM: 300691) acts as a member of the brain expressed X-linked gene family. It has been reported that BEX proteins are mainly involved in tumor growth, neurodegeneration, the cell cycle, and transcriptional regulation (19). Kim et al. suggested that the *BEX2* gene is closely related to the process and regulation of the immune response, and the expression of *BEX2* was significantly downregulated in asthmatic mouse models induced by ovalbumin, while the expression of *BEX2* was significantly upregulated and could significantly inhibit allergic airway inflammation after treatment with mesenchymal stem cell-derived extracellular vesicles (20). Previous reports documented that the remodeling of atrial structure and electrical properties was significantly associated with the dysfunction and abnormal structure of mitochondria (21, 22), that the dysfunction of mitochondria was also involved in the altered cardiac electrical properties (23, 24), and that these biological processes ultimately contribute to increased susceptibility to AF. Shao et al. proved that empagliflozin can potentially be useful in the prevention of type 2 diabetes mellitus (T2DM)-related AF by ameliorating mitochondrial dysfunction and improving atrial structure and electrical remodeling in T2DM patients (25). Furthermore, Peng et al. noticed that soybean isoflavones can activate the BNIP3/NIX pathway by upregulating the expression of *BEX2*, thereby alleviating mitochondrial dysfunction by promoting mitochondrial autophagy (26). This evidence suggests that *BEX2* may reduce the incidence of AF by mitigating mitochondrial dysfunction and inducing inflammatory or immune responses. Herein, we found that AF patients exhibited significantly reduced *BEX2* expression in comparison with SR individuals, and a positive correlation between *BEX2* expression and activated mast cells was also observed. However, these findings need to be confirmed by proteomics studies in further research.

Previous studies have found that 5-hydroxytryptamine receptor 2B (*HTR2B*) can be enriched in the calcium signaling pathway, which plays a key role in the processes of myocardial hypertrophy and remodeling (27). Due to excessive release of 5-hydroxytryptamine (5-HT) in platelets or autonomic nervous system activation, Ca^2+^ overloading may trigger AF (28, 29). As a 5-HT receptor, *HTR2B* activation significantly participates in cardiac remodeling as well as heart failure development and progression (30, 31). By impairing the deposition of collagen, blockage of *HTR2B* can suppress myocardial fibrosis (32). In addition, Nebigil et al. suggested that the overexpression of Htr2b in the mouse heart could lead to abnormal mitochondrial function of cardiac myocytes, resulting in cardiac hypertrophy (33). Furthermore, dramatically elevated expression of *HTR2B* mRNA and protein was observed in 72-h rapid electric-stimulated atrial myocytes (34). This evidence strongly suggests that *HTR2B* is significantly involved in AF development, but its association with immune cells has not been reported. In the present study, obviously increased *HTR2B* expression was observed in AF patients, and we also observed positive correlations between *HTR2B* expression and gamma delta T cells, M1 macrophages, and M2 macrophages and negative correlations with activated mast cells and resting NK cells. However, these observations need to be confirmed by further studies.

Glycosylation is an important posttranslational modification involving N-glycosylation (35) and O-glycosylation (36) and is significantly related to a variety of pathological and physiological processes. Previous studies have found that N-glycosylation is associated with the function of many ion channels, and an association between the dysfunction of glycosylation in potassium channels and long QT syndrome was observed (37). Congenital glycosylation disorder, with effects including defects in the synthesis, processing, or targeting of glycans, is a rare autosomal genetic disease (38). Almost all organs, arrhythmias, and cardiomyopathies are affected by these disorders (39). Moreover, altered glycation patterns in cardiac ion channels, such as hK_2P_17.1, may contribute to the molecular mechanisms underlying the occurrence of arrhythmogenesis correlated with glycosylation disorders (40). Polypeptide N-acetylgalactosaminyltransferase 16 (*GALNT16*), as a key gene mediating protein glycosylation, can catalyze the initial reaction in the biosynthesis of O-linked oligosaccharides and transfer N-acetyl-D-galactosamine residues to serine or threonine residues on protein receptors (41). Therefore, we surmise that the downregulation of *GALNT16* expression might lead to the occurrence of AF by causing glycosylation disorder. Moreover, *GALNT16* has been found to be significantly enriched in specific biological functions associated with protein and lipid metabolism, the AMPK signaling pathway, the prolactin signaling pathway, and the insulin/IGF pathway-protein kinase B signaling cascade (42), but the association of *GALNT16* with immune cells and AF susceptibility remains poorly understood. In the current research, we revealed that *GALNT16* expression in AF patients was dramatically decreased compared with that in normal subjects with SR, and the expression of *GALNT16* was also significantly negatively correlated with gamma delta T cells and M2 macrophages.

Recently, many studies have indicated that the inflammatory response also participates in many cardiac pathophysiological processes, such as postinfarction repair and ischaemic injury, which are characterized by immune regulation, cytokine expression, intracellular signaling pathways, and neuroendocrine system activation. Monocyte subsets are also involved in the inflammatory cascade and atherogenesis in cardiovascular disease. Elevated monocyte counts and activities are significantly related to clinical indices of chronic kidney disease (CKD) and atherosclerosis (43). In addition, the participation of other immune cells, such as neutrophils (44) and mast cells (45), was also identified in the processes of cardiovascular disease occurrence and development. A significantly increased number of CD8+ T cells in AF patients was identified by Wu et al. (46). Liu et al. showed that mast cells and neutrophils were increased in AF atrial tissue in comparison to matched SR atrial tissue (47). In addition, Liu et al. suggested that resting memory CD4 T cells and follicular helper T cells were decreased and that plasma cells, monocytes, resting dendritic cells, and neutrophils were increased in AF samples compared with SR samples (48). Nevertheless, different patterns of immune cell infiltration in AF were observed in the current study compared with previous studies. Our results suggested decreased infiltration of regulatory T cells (Tregs) and activated mast cells and increased infiltration of gamma delta T cells, M2 macrophages, and resting mast cells in AF patients compared with SR subjects. In addition, we also revealed the interactions among these 22 types of infiltrated immune cells in AF. We noticed that activated mast cells were negatively associated with resting mast cells; Tregs were negatively associated with M1 macrophages and positively associated with activated NK cells; M2 macrophages were negatively associated with resting dendritic cells, eosinophils, resting memory CD4 T cells, and monocytes; CD8 T cells were negatively associated with resting memory CD4 T cells; memory B cells were positively associated with plasma cells. These results suggested that there may be complex immune regulation mechanisms governing the occurrence and development of AF. However, the potential mechanisms of these correlations between infiltrated immune cells need to be verified by experiments *in vivo* and *in vitro*.

This research had several limitations. First, the validation samples included in the current research were recruited from only a single center with small sample sizes. It is unclear whether the expression levels of the key genes differ among individuals in different regions or races. Therefore, the results of this study need to be further tested in multicentre studies and using larger sample sizes. Second, although we found that the expression trends of key genes in blood samples of patients with AF were consistent with the bioinformatics prediction results based on left atrial samples, the expression trends of key genes still need to be further verified in other left atrial samples. Third, more *in vivo* and *in vitro* studies are needed to clarify the underlying mechanisms of these correlations between *GALNT16, HTR2B, RAB8A*, and *BEX2* as well as infiltrating immune cells in AF.

## Conclusions

In summary, we determined that *BEX2, HTR2B*, and *GALNT16* may become potential diagnostic markers or novel therapeutic targets in AF. We noticed that resting mast cells, M2 macrophages, and gamma delta T cells might be involved in AF initiation; however, activated mast cells, CD8 T cells, and regulatory T cells (Tregs) may serve protective roles in AF. The interaction mechanisms between *BEX2, HTR2B*, and *GALNT16* and immune cells may be of great significance to the pathogenesis and progression of AF.

## Data availability statement

The original contributions presented in the study are included in the article/Supplementary material, further inquiries can be directed to the corresponding authors.

## Ethics statement

The study design was approved by the Ethics Committee of Hunan Provincial People's Hospital (No: LL-20210615-144). The patients/participants provided their written informed consent to participate in this study.

## Author Contributions

P-FZ conceived the study, participated in the design, performed the statistical a2nalyses, and drafted the manuscript. Z-YL and HWP conceived the study, participated in the design, and helped to draft the manuscript. Z-YL carried out the epidemiological survey and collected the samples. L-ZC and PL performed the statistical analyses. All authors read and approved the final manuscript.

## Funding

This study was supported by the Key Research and Development Program of Hunan Province (No: 2019SK2021) and the Natural Science Foundation of Hunan Province (NO:2020JJ4406). There was no role of the funding body in the design of the study and collection, analysis, and interpretation of data and in writing the manuscript.

## Conflict of interest

The authors declare that the research was conducted in the absence of any commercial or financial relationships that could be construed as a potential conflict of interest.

## Publisher's note

All claims expressed in this article are solely those of the authors and do not necessarily represent those of their affiliated organizations, or those of the publisher, the editors and the reviewers. Any product that may be evaluated in this article, or claim that may be made by its manufacturer, is not guaranteed or endorsed by the publisher.
