# Supplementary material for: Integrative identification of immune-related key genes in atrial fibrillation using weighted gene coexpression network analysis and machine learning
Source: Front Cardiovasc Med. 2022 Jul 27;9:922523. doi: 10.3389/fcvm.2022.922523 (PMC9363882; doi:10.3389/fcvm.2022.922523)
Supplement: Supplementary file 1 [file Data_Sheet_1.docx]

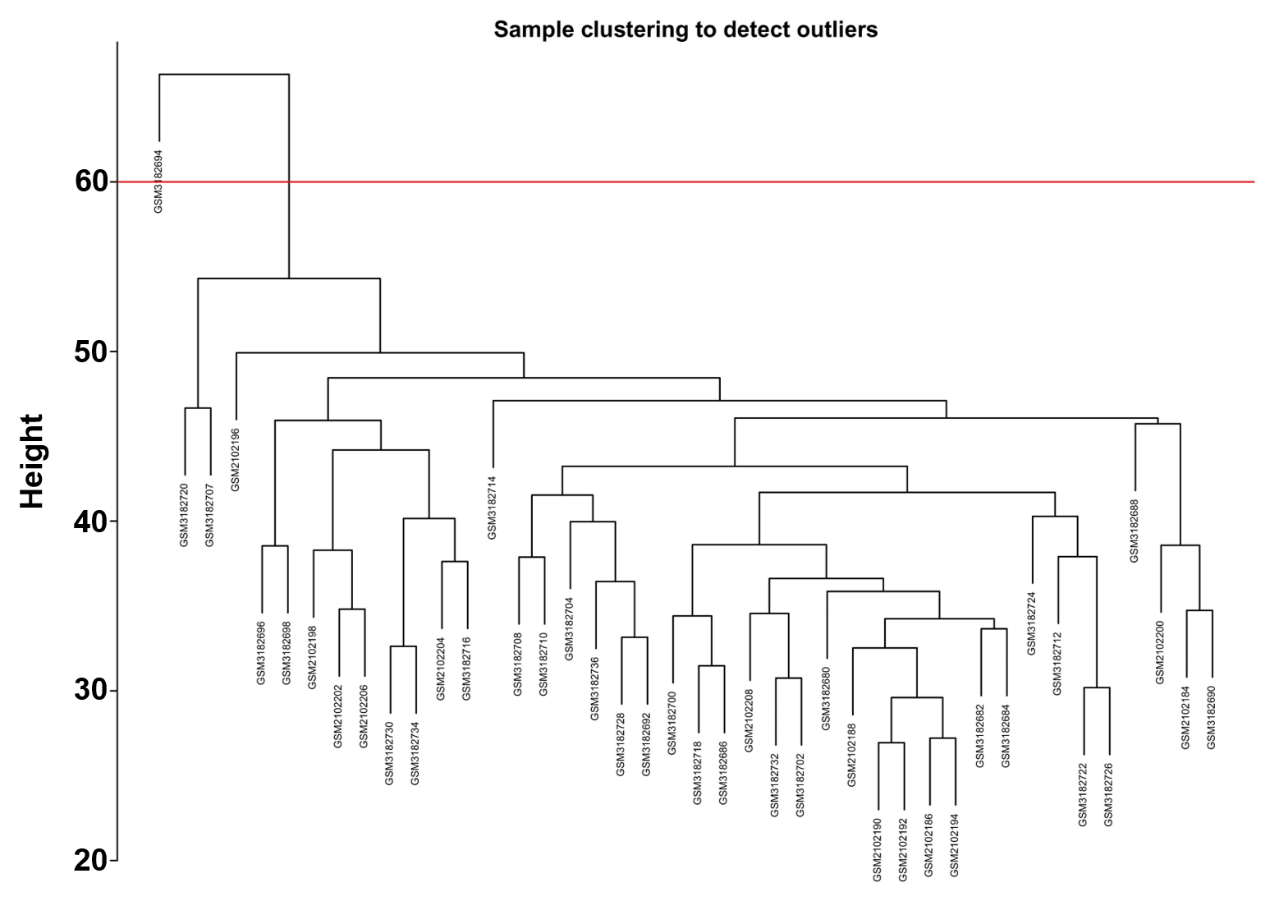


**Additional** **Figure S1: Clustering dendrogram of samples.**

Cut height = 60 was used to divide 42 samples into two different cluster types. Cluster 1 contains the following samples (GSM2102198, GSM2102200, GSM2102202, GSM2102204, GSM2102206, GSM2102208, GSM2102184, GSM2102186, GSM2102188, GSM2102190, GSM2102192, GSM2102194, GSM2102196, GSM3182708, GSM3182710, GSM3182712, GSM3182714, GSM3182716, GSM3182718, GSM3182720, GSM3182722, GSM3182724, GSM3182726, GSM3182728, GSM3182730, GSM3182732, GSM3182734, GSM3182736, GSM3182680, GSM3182682, GSM3182684, GSM3182686, GSM3182688, GSM3182690, GSM3182692, GSM3182696, GSM3182698, GSM3182700, GSM3182702, GSM3182704, GSM3182707), cluster 2 contains the following samples (GSM3182694)


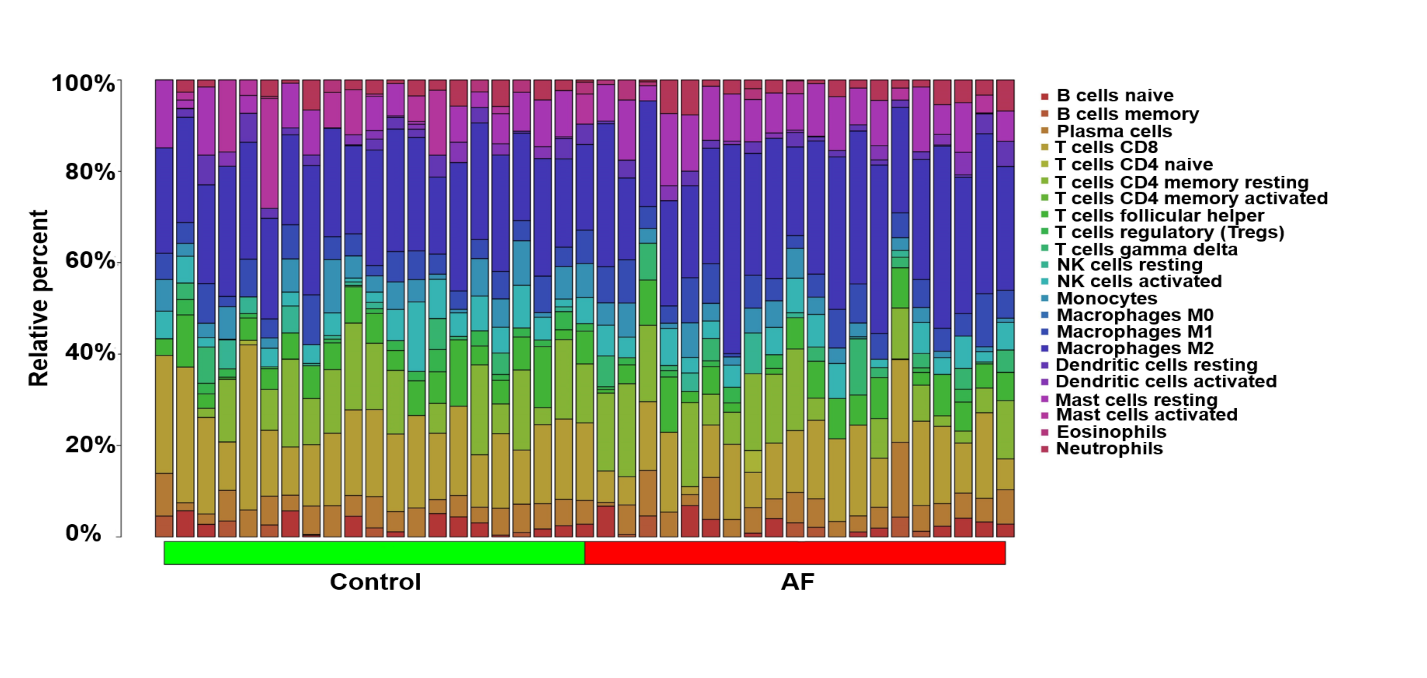
 **Additional Figure S2: Infiltration pattern of immune cell subtypes in validation set.**

The bar plot visualizing the relative percent of 22 immune cell in each sample.
